# Correction: Sex differences in pulmonary function parameters among Indigenous Australians with and without chronic airway disease

**DOI:** 10.1371/journal.pone.0307697

**Published:** 2024-07-18

**Authors:** Subash S. Heraganahally, Timothy Howarth, Lisa Sorger, Helmi Ben Saad

In Table 3, the mean values and 95% confidence intervals for all columns are incorrect. Please see the correct Table 3 here.

**Table 3:** Pulmonary function tests parameters according to presence of chronic airway disease radiological findings.

Radiology findings		Without chronic airway disease			With chronic airway disease		
Sex		Female (n = 179)	Male (n = 111)	p-value	Female (n = 92)	Male (n = 103)	p-value
**FVC**	**LLN (L)**	2.65 (2.59, 2.71)	3.73 (3.63, 3.83)	<0.001*	2.49 (2.4, 2.58)	3.45 (3.35, 3.55)	<0.001*
	**Pre (L)**	2.13 (2.04, 2.23)	3.09 (2.93, 3.24)	<0.001*	1.77 (1.65, 1.88)	2.43 (2.29, 2.58)	<0.001*
	**Pre (%)**	64.05 (61.68, 66.42)	67.01 (64.26, 69.76)	0.116	56.08 (52.74, 59.41)	56.51 (53.42, 59.61)	0.848
	**Post (L)**	2.19 (2.1, 2.28)	3.14 (2.98, 3.29)	<0.001*	1.87 (1.75, 1.98)	2.56 (2.42, 2.71)	<0.001*
	**Post (%)**	65.69 (63.43, 67.95)	67.45 (64.42, 70.48)	0.353	59.32 (56.11, 62.52)	58.8 (55.59, 62)	0.821
	**BDR (% change)**	3.47 (2.05, 4.9)	1.96 (0.49, 3.44)	0.166	7.95 (3.93, 11.96)	6.45 (4.78, 8.11)	0.477
**FEV**_**1**_	**LLN (L)**	2.09 (2.03, 2.14)	2.9 (2.82, 2.99)	<0.001*	1.94 (1.87, 2.02)	2.64 (2.56, 2.72)	<0.001*
	**Pre (L)**	1.65 (1.56, 1.74)	2.35 (2.2, 2.5)	<0.001*	1.19 (1.08, 1.3)	1.5 (1.37, 1.64)	0.001*
	**Pre (%)**	60.72 (57.93, 63.5)	63.85 (60.36, 67.33)	0.168	47.48 (43.58, 51.38)	44.34 (40.69, 47.99)	0.244
	**Post (L)**	1.73 (1.64, 1.81)	2.44 (2.29, 2.59)	<0.001*	1.28 (1.16, 1.39)	1.61 (1.47, 1.75)	<0.001*
	**Post (%)**	63.59 (60.87, 66.31)	66.16 (62.68, 69.65)	0.250	50.73 (46.72, 54.73)	47.57 (43.85, 51.29)	0.253
	**BDR (% change)**	6.06 (4.48, 7.63)	4.63 (2.84, 6.42)	0.251	8.34 (5.42, 11.25)	8.09 (5.95, 10.23)	0.890
**FEV**_**1**_ **/FVC**	**LLN (absolute)**	0.71 (0.69, 0.73)	0.69 (0.69, 0.7)	0.070	0.7 (0.69, 0.7)	0.68 (0.67, 0.69)	<0.001*
	**Pre (absolute)**	0.76 (0.75, 0.78)	0.75 (0.73, 0.78)	0.453	0.66 (0.63, 0.69)	0.6 (0.57, 0.63)	0.004*
	**Pre (%)**	93.72 (91.55, 95.89)	95.05 (92.14, 97.97)	0.463	82.9 (78.97, 86.83)	76.64 (73.08, 80.2)	0.020*
	**Post (absolute)**	0.78 (0.76, 0.79)	0.77 (0.75, 0.79)	0.563	0.67 (0.64, 0.7)	0.61 (0.58, 0.64)	0.004*
	**Post (%)**	95.84 (93.7, 97.97)	97.31 (94.56, 100.05)	0.403	83.58 (79.62, 87.54)	77.98 (74.23, 81.73)	0.043*
Data were reported as mean (95% CI). p-values derived from 2-tailed Students t-test. *Denotes statistically significant association (p<0.05). **Abbreviations**: **BDR**, Bronchodilator responsiveness; **FEV**_**1**_, Forced expiratory volume in one second; **FVC**, Forced vital capacity; **LLN**, Lower limit of normal; **Post**: Post bronchodilator, **Pre**, Pre bronchodilator; **%**, percentage of predicted value.							
