# Supplementary material for: Sex differences in pulmonary function parameters among Indigenous Australians with and without chronic airway disease
Source: PLoS One. 2022 Feb 8;17(2):e0263744. doi: 10.1371/journal.pone.0263744 (PMC8824342; doi:10.1371/journal.pone.0263744)
Supplement: Attachment — Submitted filename: PFT-Sex-Response to Reviewer comments.docx [file pone.0263744.s001.docx]

Darwin, Australia.

Date: 31/12/2021

To the

Academic Editor

PLOS ONE

Dear Dr James West

Thank you for inviting and giving us an opportunity to revise and resubmit our original manuscript entitled ***“Sex differences in pulmonary function parameters among Indigenous Australians with and without chronic airway disease”*** to your esteemed journal. **(PONE-D-21-26363)**

We sincerely appreciate the reviewers’ time, comments and suggestions regarding our study. We have made changes to the revised manuscript taking into consideration of the reviewers’ suggestions and comments, which are detailed below.

**Response to reviewers comments.**

**Reviewer: 1**

**Comments to the Author**

Reviewer #1: the author performed a retrospective study on 485 adults to assess Sex differences in pulmonary function parameters among Indigenous Australians with and without chronic airway disease and concluded that There are significant sex differences in the PFTPs in Indigenous Australians. Recognizing these differences may be of value in the accurate diagnosis, management, monitoring and prognostication of CADs in this population. the topic is important and the manuscript is well designed and written but i have few comments:
**Response:** We sincerely thank and very pleased for the reviewer above opinion and comments. Especially for the comment that ***“the topic is important and the manuscript is well designed and written”***

1- the manuscript needs good English editing

**Response:** We thank the reviewer for bring this to our attention. We have addressed this in the revised manuscript were required and appropriate.

2- what were the inclusion and the exclusion criteria?

**Response:** We thank the reviewer for asking us to clarify regarding this. We have addressed this in the revised manuscript and reads as in the revised manuscript under the method section

Study participants – Inclusion and exclusion criteria

Patients who underwent PFTs and had a chest radiography available (Chest X-Ray and computed tomography (CT)) to assess the presence/absence of any radiological pulmonary abnormalities (specifically demonstrating radiographic evidence of COPD/emphysema, bronchiectasis or a combination of both COPD & bronchiectasis) were included in the analysis. Patients PFTs that were assessed not acceptable for session quality were excluded.

3- how did you estimate the sample size for this study? what was the power of the study?

**Response:** We thank the reviewer for asking us to clarify regarding this. We have added a subsection entitled “sample size” .

As it was a retrospective study, we did not calculate sample size.

In the revised manuscript under the subheading - Statistical analysis it reads as

“As the study was a retrospective study no prior sample size estimation was conducted, however, post-hoc power analysis was conducted for testing of mean differences between females and males for pre- bronchodilator values of FVC, FEV1 and FEV1/FVC (Table1).”

However, we did calculate a sample size for this study during our revision as per the reviewer suggestion as detailed below, but did not included in the revised manuscript. The authors are in the opinion that the above statement may be appropriate for this study. If the reviewer like us to include the below statement, we are happy to consider.

“Given the pioneer character of our study, these two values were obtained from a comparative study of PFTPs in healthy male (n=75) and female (n=75) subjects (age range: 20-75 years) of western Rajasthan in India 9, where FEV1/FVC means±SD of females and males were, respectively, 0.8780±0.0818 and 0.8516±0.0813 (ie; a common standard-deviation equal to 0.0815).

The sample size for the study was 407 (230 females and 177 males). The assumption of a percentage of 70% for exclusion criteria gave a revised sample of 1357 patients [= 407/(1-0.70)].”

We hope that we have addressed the reviewers comments and suggestion satisfactorily and are acceptable to the reviewer and the editor.

We eagerly look forward on the decision for consideration for publication of this revised manuscript.

We once again thank the editor for giving us the opportunity and the reviewer for dedicating their valuable time to review our study.

Thanking you,

Sincerely,

On behalf of all authors

Corresponding author

Assoc/Prof. HSS

----------------------------------------------------------------------------------------------------------------
